# Corrigendum to “Predictors of Loss of Ambulation in Duchenne Muscular Dystrophy: A Systematic Review and Meta-Analysis”

**DOI:** 10.1177/22143602251320076

**Published:** 2025-02-27

Landfeldt E, Alemán A, Abner S, Zhang R, Werner C, Tomazos I, et al. Predictors of Loss of Ambulation in Duchenne Muscular Dystrophy: A Systematic Review and Meta-Analysis. *J Neuromuscul Dis* 2024; 11(3): 579-612. DOI: 10.3233/JND-230220

In above mentioned systematic review, the authors erroneously stated in the summary of the evidence under the Discussion section that mutations amenable to exon 53 skipping are associated with later loss of ambulation (LoA).

The following text “In our systematic review, we found evidence that deletion of exons 3–7 [17, 49], proximal mutations (upstream intron 44) [31], single exon 45 deletions [49], and mutations amenable of skipping exon 8 [24, 49], exon 44 [13, 17, 24, 45, 49], and exon 53 [41], were associated with later loss of ambulation in DMD. On the other hand, distal mutations (intron 44 and downstream) [31], deletion of exons 49–50 [49], and mutations amenable of skipping exon 45 [24], and exon 51 [24, 49] were related with earlier loss of ambulation in DMD patients.” has been corrected and should be read as:

In our systematic review, we found evidence that deletion of exons 3–7 [17, 49], proximal mutations (upstream intron 44) [31], single exon 45 deletions [49], and mutations amenable of skipping exon 8 [24, 49], and exon 44 [13, 17, 24, 45, 49], were associated with later loss of ambulation in DMD. On the other hand, distal mutations (intron 44 and downstream) [31], deletion of exons 49–50 [49], and mutations amenable of skipping exon 45 [24], exon 51 [24, 49], and exon 53 [41], were related with earlier loss of ambulation in DMD patients.

This error was also present in Figure 4 and Figure 5. The corrected figures are given below: 
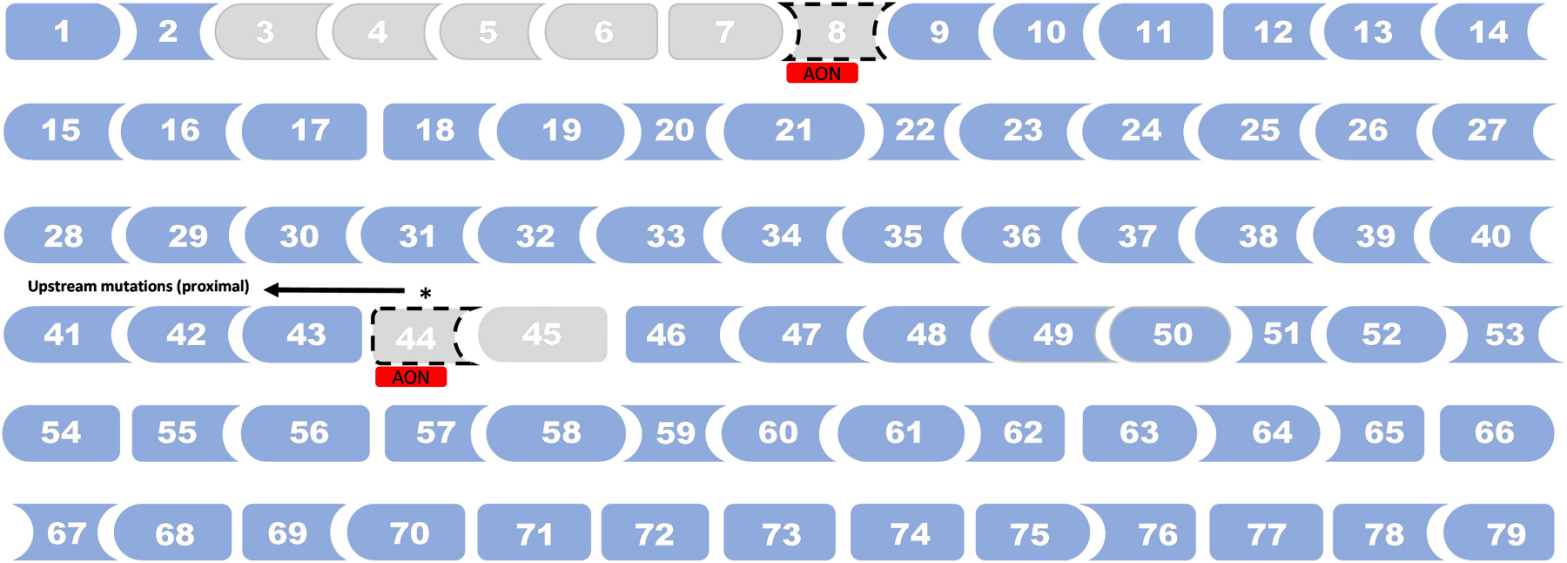


Fig. 4. *DMD* mutations spots associated with later loss of ambulation in DMD.


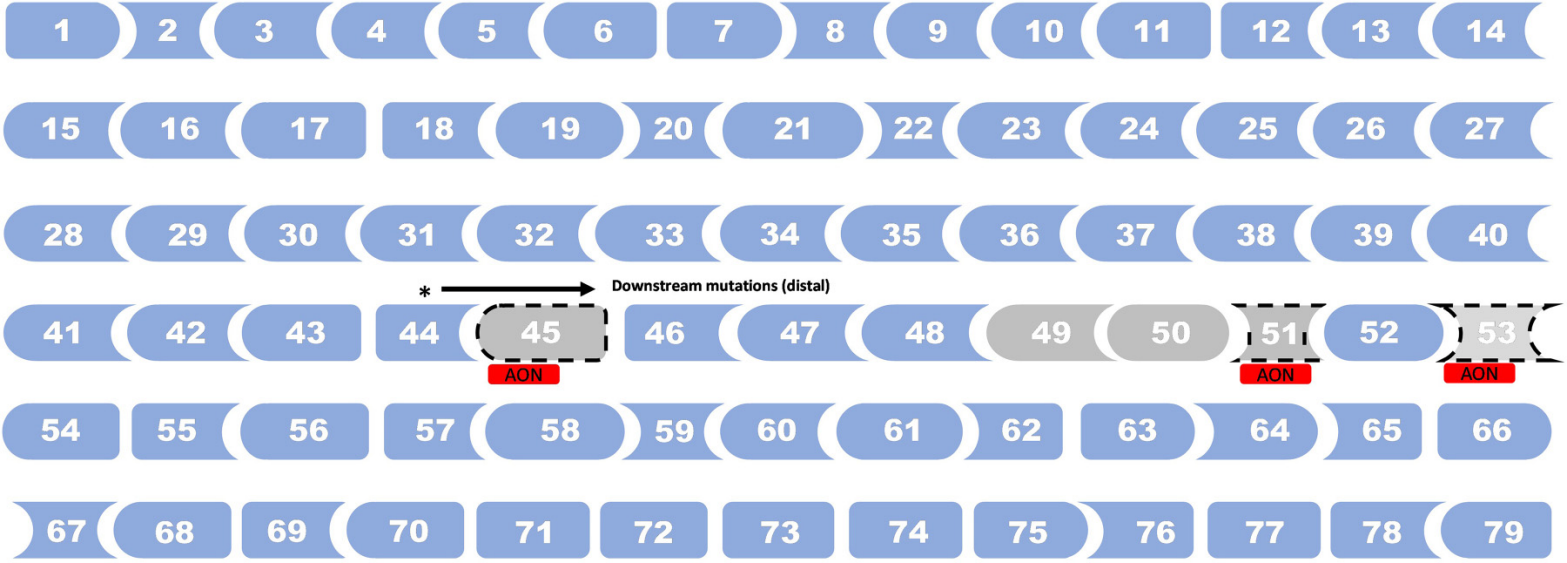


Fig. 5. *DMD* mutations spots associated with earlier loss of ambulation in DMD.

The online version has been updated to reflect the changes.

